# Multimodal Nutraceutical and Psychological Intervention for GGT Reduction in Individuals with Alcohol Use Disorder

**DOI:** 10.3390/nu18111676

**Published:** 2026-05-23

**Authors:** Nilca Stefania Diana, Tarcea Monica, Gliga Florina

**Affiliations:** 1 Doctoral School, George Emil Palade University of Medicine, Pharmacy, Science and Technology of Targu Mures, 540142 Târgu Mureș, Romania; 2 Department of Community Nutrition and Food Safety, George Emil Palade University of Medicine, Pharmacy, Science and Technology of Targu Mures, 540142 Târgu Mureș, Romania; 3 Department of Physiopathology, George Emil Palade University of Medicine, Pharmacy, Science and Technology of Targu Mures, 540142 Târgu Mureș, Romania

**Keywords:** gamma-glutamyl transferase, nutraceuticals, psychological intervention, alcohol abstinence, alcohol use disorder

## Abstract

Background: Elevated gamma-glutamyl transferase (GGT) is a biomarker associated with alcohol-related hepatic stress and oxidative imbalance. Although alcohol abstinence is the primary determinant of GGT normalization, adjunctive strategies may support biochemical improvement in real-world settings. Methods: This non-randomized cohort study included 197 of 1957 screened participants (10.1%), stratified according to baseline GGT into 55–99 U/L (*n* = 95) and ≥100 U/L (*n* = 102). Participants in the higher baseline subgroup underwent a multimodal intervention consisting of nutraceutical supplementation (silymarin, essential phospholipids, and a polyherbal antioxidant formulation) combined with structured psychological support aimed at promoting alcohol abstinence. The primary outcome was the change in GGT between baseline (T1) and follow-up (T2). Secondary outcomes included the proportion of participants achieving GGT reduction and the magnitude of change according to baseline severity. Clinical trial registration: ClinicalTrials.gov Identifier: NCT07603726. Results: Among participants with baseline GGT ≥ 100 U/L, GGT levels decreased from a median of 133.73 to 97.41 U/L (*p* < 0.001), whereas in the 55–99 U/L subgroup, median GGT changed from 67.49 to 66.51 U/L without reaching statistical significance (*p* = 0.072). Participants in the higher baseline subgroup demonstrated greater GGT reductions (median ΔGGT: −35.25 vs. −2.58 U/L), a higher proportion achieving GGT reduction (91.2% vs. 70.5%), and higher odds of GGT reduction at follow-up in exploratory analysis (OR = 4.32, 95% CI: 1.91–9.75). Conclusions: In this real-world cohort, reductions in GGT levels were observed, particularly among individuals with elevated baseline values (≥100 U/L) who underwent the multimodal intervention. These findings support monitoring GGT dynamics in routine clinical practice, where GGT remains a practical and accessible biomarker due to its widespread availability, low cost, and sensitivity to oxidative and alcohol-related hepatic stress.

## 1. Introduction

Gamma-glutamyl transferase (GGT) is a key enzyme involved in glutathione metabolism and antioxidant defense, widely used as a clinical biomarker of hepatobiliary dysfunction [1]. Elevated serum GGT levels have been associated with increased mortality risk and a wide range of diseases, including cerebrovascular disease and cancer [2,3,4]. However, GGT is a non-specific marker influenced by multiple metabolic and systemic conditions, including obesity, diabetes, and medication use, which limits its diagnostic accuracy as a standalone indicator of alcohol-related hepatic disease [1,3].

Liver status and hepatocellular injury are commonly assessed using serum biomarkers such as alanine aminotransferase (ALT), aspartate aminotransferase (AST) and GGT, the latter being particularly sensitive to alcohol-related hepatic stress and oxidative imbalance [5,6,7].

More specific alcohol biomarkers, such as carbohydrate-deficient transferrin (CDT) and phosphatidylethanol (PEth), may improve the accuracy of alcohol use assessment, but are not consistently available in routine clinical practice, where conventional markers such as GGT remain widely used due to accessibility and cost-effectiveness [5,6,8,9,10].

Alcohol use disorder (AUD), as defined by standardized diagnostic frameworks such as the DSM-5 and the ICD-10, contributes substantially to the global burden of disease and premature mortality [11,12,13,14]. Across all severity levels, alcohol abstinence remains the cornerstone of management, while pharmacological and nutraceutical interventions are considered adjunctive rather than primary therapeutic strategies [15,16,17].

In clinical practice, alcohol-related liver disease is commonly evaluated using liver function tests, with elevated aminotransferases, an AST/ALT ratio > 2, and increased GGT frequently observed in individuals with AUD [5,6,7,16].

Nutraceutical and pharmacological approaches, including antioxidants, omega-3 fatty acids, and herbal compounds, may support hepatic function through antioxidant and inflammatory mechanisms [17,18]. However, current evidence does not support their independent efficacy in modifying long-term clinical outcomes in alcohol-related liver disease [5,15]. Current guidelines emphasize that abnormal liver function tests should be interpreted in the context of clinical history, metabolic risk factors, and potential alcohol exposure [19].

Although no universally accepted GGT cutoff exists, markedly elevated levels have been associated with adverse liver-related and cardiometabolic outcomes, increased mortality, and greater likelihood of alcohol-related pathology [2,3,20,21]. Psychological counseling is increasingly recognized as an important complementary approach in AUD management, supporting behavioral modification, sustained alcohol abstinence, and treatment adherence [22,23,24,25,26,27,28,29]. Current pharmacological strategies for AUD include relapse-prevention and craving-reduction approaches, although their implementation varies across healthcare systems [27]. Alcohol-related liver injury involves multiple pathophysiological mechanisms, including oxidative stress, inflammation, and disruption of the gut–liver axis, which together contribute to hepatic dysfunction in chronic alcohol exposure [15,18,30]. Beyond alcohol-related disease, elevated GGT has been associated with adverse metabolic, cardiovascular, and prognostic outcomes, reinforcing its broader clinical relevance as an accessible biomarker in routine practice [2,3,4,31].

Chronic psychological distress and behavioral self-regulation mechanisms may also influence alcohol-related behaviors, treatment adherence, and relapse risk [32,33]. Alcohol-associated liver disease represents a major contributor to the global burden of liver disease, and its management increasingly supports integrated multidisciplinary approaches addressing both hepatic dysfunction and alcohol-related behavioral challenges [34,35,36]. Because GGT may also be influenced by metabolic liver disease and related comorbidities, interpretation of biochemical changes requires caution [37]. Behavioral frameworks further support individualized psychological approaches in alcohol use disorder [22,23,24,25,26,38].

For the purposes of this study, a threshold of ≥100 U/L was applied as a pragmatic cutoff for stratifying participants according to baseline biochemical severity, rather than as a diagnostic threshold.

The present study aimed to explore the association between a multimodal intervention combining nutraceutical supplementation with structured psychological support and changes in GGT levels in individuals with alcohol use disorder.

## 2. Materials and Methods

This study was designed as a non-randomized interventional cohort study conducted in a real-world clinical setting. Participants were recruited from individuals attending routine medical and psychological evaluations at a single center in Targu Mures, Romania between 2024 and 2026.

Eligible subjects were adults (≥18 years) presenting with elevated gamma-glutamyl transferase (GGT) levels above the reference threshold (>55 U/L) and clinical features consistent with alcohol use disorder (AUD), assessed according to the Diagnostic and Statistical Manual of Mental Disorders, Fifth Edition (DSM-5), through clinical interviews and psychological questionnaires. Alcohol Use Disorders Identification Test (AUDIT) was used as a supportive screening instrument. The baseline distribution of AUD severity across GGT subgroups is presented in Supplementary Table S2. Patients with chronic inflammatory diseases, advanced non-alcohol-related liver disease, severe comorbidities, or incomplete data were excluded.

GGT was selected as the primary hepatic biomarker due to its wide availability, low cost, and inclusion in mandatory medical examinations under Romanian Regulation No. 579/2025, supporting its applicability in occupational health screening and real-world clinical practice [39]. Serum GGT levels were measured using a spectrophotometric method on a JEOL JCA-BM 6010/C analyzer (JEOL Ltd., Tokyo, Japan).

**Figure 1:**
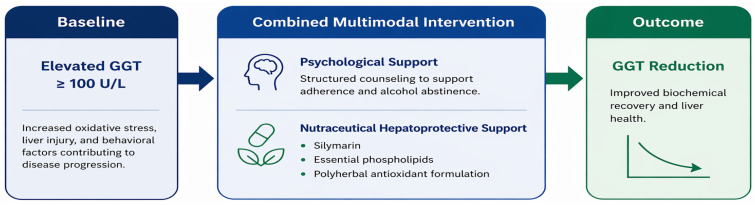
Integrated nutraceutical and psychological intervention framework and its components in individuals with alcohol use disorder.

The multimodal intervention framework is illustrated in Figure 1.

Of the 1957 participants assessed during routine clinical evaluations between 2024 and 2026, 197 (10.1%) presented with GGT levels above the upper reference limit and were eligible for inclusion. Among these, 194 were male (98.48%) and 3 were female (1.52%), all of whom provided informed consent and were included in the study. The sample was predominantly male, reflecting the occupational characteristics of the study population, which should be considered when interpreting the generalizability of the findings.

The 197 participants had a median age of 49 years (IQR: 39–55). Participants were stratified according to baseline GGT levels into two predefined subgroups. Participants with moderately elevated GGT values (55–99 U/L) included 95 individuals, with a median baseline GGT of 67.49 U/L (IQR: 60.37–78.45) and a median age of 46 years (IQR: 39.5–53). Participants with higher baseline GGT levels (≥100 U/L) included 102 individuals, with a median baseline GGT of 133.73 U/L (IQR: 114.78–168.86) and a median age of 51 years (IQR: 39.5–56).

The 100 U/L cutoff was selected as a pragmatic marker of marked biochemical elevation for exploratory stratification. It was not intended as a diagnostic threshold, but rather to explore associations across baseline GGT categories [19,20,21].

Participants with baseline GGT ≥ 100 U/L received a physician-supervised nutraceutical intervention including silymarin (150 mg twice daily), essential phospholipids (300 mg three times daily), and a polyherbal antioxidant formulation (two tablets twice daily), administered orally according to manufacturer recommendations. Structured weekly psychological support was delivered by a licensed clinical psychologist and included motivational counseling, psychoeducation regarding alcohol-related harm, and guidance on lifestyle modification to promote alcohol abstinence and behavioral change. Participants with baseline GGT levels between 55–99 U/L received routine care consisting of general advice regarding alcohol abstinence. Details regarding the nutraceutical products used in the intervention are provided in Supplementary Table S1.

Data were analyzed using JASP (Version 0.19.1) and data preprocessing was performed using Microsoft Excel (Microsoft Corp., Redmond, WA, USA). All statistical tests were two-tailed, and statistical significance was set at α = 0.05. Continuous variables were first assessed for normality using the Shapiro–Wilk test. Given the non-normal distribution of GGT levels, nonparametric statistical methods were applied. Paired comparisons were performed using the Wilcoxon signed-rank test, and effect size was calculated using the rank-biserial correlation coefficient (r). The proportion of participants achieving GGT reduction at follow-up was compared between baseline GGT subgroups, and odds ratios (ORs) with 95% confidence intervals were calculated as exploratory measures of association. Given the non-randomized design, intervention allocation according to baseline GGT category, and marked differences in alcohol use disorder severity between subgroups, these analyses were interpreted as exploratory and descriptive rather than causal. Time 1 (T1) corresponded to baseline, whereas Time 2 (T2) represented follow-up evaluation.

Follow-up assessments were conducted at variable intervals reflecting routine clinical practice, consistent with the inherent variability of behavioral follow-up in real-world alcohol use disorder management.

This study was retrospectively registered at ClinicalTrials.gov (Identifier: NCT07603726), following clarification that its real-world interventional clinical practice design met clinical trial registration criteria. The study was conducted in accordance with the Declaration of Helsinki and approved by the Ethics Committee of Medical Center TOPMED, Targu Mureș, Romania (protocol code 1/4 January 2024), as well as by the Ethics Committee of George Emil Palade University of Medicine, Pharmacy, Science and Technology of Targu Mureș, Romania (UMFST) (protocol code 3908/17 December 2025).

## 3. Results

The study population was stratified into two predefined baseline GGT subgroups: 55–99 U/L (*n* = 95; median GGT at T1 was 67.49 U/L [IQR 60.37–78.45]) and ≥100 U/L (*n* = 102; median GGT at T1 was 133.73 U/L [IQR 114.78–168.86]). Baseline characteristics of the study population are presented in Table 1. Baseline alcohol use disorder severity differed markedly between baseline GGT subgroups (χ^2^ (2) = 120.08, *p* < 0.001; Supplementary Table S2).

Within-subgroup paired analyses revealed different patterns of GGT change over time. In the 55–99 U/L subgroup (*n* = 95), median GGT levels decreased slightly from 67.49 U/L at T1 to 66.51 U/L (IQR: 53.56–77.91) at T2 (Wilcoxon signed-rank test: *p* = 0.072), with a small effect size (r = 0.18), whereas participants in the ≥100 U/L subgroup (*n* = 102) showed a significant reduction over time, with a large effect size (r = 0.80), as summarized in Table 2.

Wilcoxon signed-rank test. Individual GGT trajectories for each subgroup are presented in Supplementary Figures S2 and S3.

Changes in GGT levels (ΔGGT = T2 − T1) were examined across subgroups. The median ΔGGT was −2.58 U/L in the 55–99 U/L subgroup and −35.25 U/L in the ≥100 U/L subgroup, as presented in Table 3.

The distribution of ΔGGT values is presented in Supplementary Figure S1.

**Figure 2:**
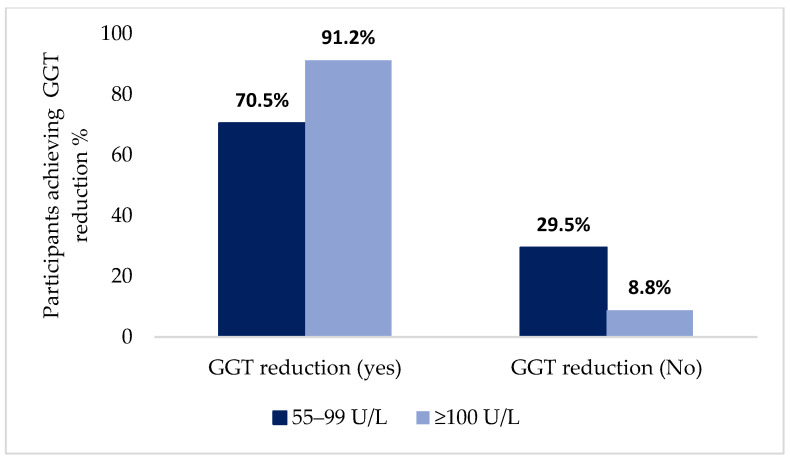
Proportion of participants achieving GGT reduction according to baseline GGT subgroups.

The proportion of participants achieving GGT reduction was 70.5% in the 55–99 U/L subgroup (67/95; 95% CI: 60.7–78.8%) and 91.2% in the ≥100 U/L subgroup (93/102; 95% CI: 84.1–95.3%). An exploratory analysis of GGT reduction proportions across baseline GGT categories yielded an odds ratio of 4.32 (95% CI: 1.91–9.75; *p* < 0.001), as presented in Table 4 and Figure 2.

**Figure 3:**
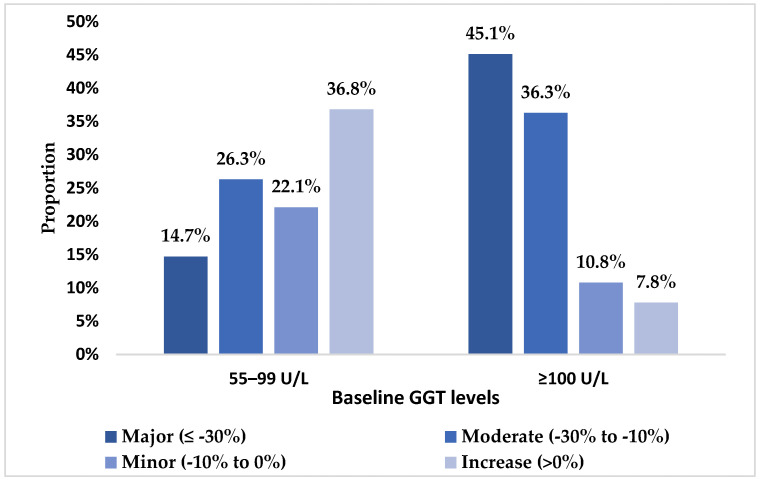
Distribution of GGT response patterns (major, moderate, minor, increase) across baseline GGT subgroups.

Response patterns were examined across baseline GGT subgroups. In the ≥100 U/L subgroup, 81% of participants showed major or moderate reductions in GGT levels, whereas 41% of participants in the 55–99 U/L subgroup showed major or moderate reductions in GGT levels. Increases in GGT levels were more frequently observed in the lower baseline subgroup (Figure 3).

## 4. Discussion

The present study explored biochemical changes in GGT levels in individuals with alcohol use disorder managed with a multimodal intervention combining nutraceutical support and structured psychological counseling in a real-world clinical setting. Greater reductions in GGT were observed among participants with higher baseline values, whereas changes in those with lower baseline GGT were limited and did not reach statistical significance.

Exploratory analysis yielded an odds ratio of 4.32 (95% CI: 1.91–9.75) for GGT reduction in the ≥100 U/L baseline subgroup; however, this finding should be interpreted cautiously, as subgroup allocation was determined according to baseline GGT category within a non-randomized design, and alcohol use disorder severity differed substantially between groups.

Alcohol abstinence is a well-established determinant of GGT normalization and likely the main driver of the observed biochemical changes. The structured psychological support provided may have facilitated adherence to abstinence-related behaviors and promoted healthier lifestyle patterns associated with improved liver outcomes, thereby contributing indirectly to the observed biochemical changes [22,23,24,25,26,30,39].

Previous studies have reported reductions in GGT levels following nutraceutical interventions such as silymarin, particularly when combined with lifestyle modification. Randomized and prospective studies suggest a potential supportive effect on biochemical liver markers [40,41,42]. Proposed mechanisms include the antioxidant and membrane-stabilizing properties of silymarin and related compounds, which may contribute to reduced oxidative stress and enhance hepatocellular integrity [40,41,42]. Essential phospholipids have also been investigated as adjunctive hepatoprotective agents in chronic liver disease settings [40].

A notable feature of the present study was the evaluation of an integrated multimodal approach in a clinically relevant real-world population.

Despite the limitations inherent to its non-randomized real-world design, the study provides clinical data suggesting that integrated approaches combining behavioral support with adjunctive strategies may be associated with improvements in biochemical markers in individuals with alcohol use disorder. These findings may help generate hypotheses for future prospective controlled studies.

Future research should prioritize randomized controlled designs, standardized follow-up intervals, and objective biomarkers of harmful drinking. Comparable study groups and clearly defined intervention allocation would help reduce bias related to baseline differences. Inclusion of larger and more diverse populations would improve generalizability. Study designs allowing separate evaluation of psychological and nutraceutical components would also clarify the relative contribution of each intervention modality.

### Limitations

The present study has several limitations. First, the non-randomized interventional design limits causal inference, and comparisons between baseline GGT subgroups should be interpreted cautiously because the groups were not clinically equivalent. Alcohol use disorder severity differed significantly between GGT categories (χ^2^ (2) = 120.08, *p* < 0.001; Supplementary Table S2), and the greater reductions observed in participants with higher baseline GGT levels may also have been partially influenced by regression to the mean.

Second, GGT was used as the primary biomarker, although it is a sensitive but non-specific indicator of hepatic dysfunction that may be influenced by metabolic and systemic comorbidities. The absence of alcohol-specific biomarkers such as carbohydrate-deficient transferrin (CDT) or phosphatidylethanol (PEth) limited objective assessment of changes in alcohol consumption over time. Third, the integrated intervention precluded disentangling the independent contribution of the psychological and nutraceutical components.

Follow-up assessments were also conducted at variable time intervals, which may have introduced heterogeneity in biochemical responses, consistent with real-world clinical practice. Finally, the single-center design and predominantly male study population limit the generalizability of the findings.

## 5. Conclusions

In this real-world clinical study, reductions in GGT levels were observed over time, particularly among individuals with higher baseline values (≥100 U/L) who underwent the intervention. These findings support monitoring GGT dynamics in alcohol use disorder and may help generate hypotheses for future controlled studies evaluating integrated multimodal interventions.

## Figures and Tables

**Table 1:** Baseline characteristics of the study population.

Variable	Subgroup 55–99 U/L	Subgroup ≥ 100 U/L
Age (median, IQR)	46 (39.5–53)	51 (39.5–56)
GGT T1 (median, IQR)	67.49 (60.37–78.45)	133.73 (114.78–168.86)

**Table 2:** Within-subgroup changes in GGT.

Baseline GGT Subgroup (U/L)	*n*	Median T1	Median T2	*p*-Value	Wilcoxon Z	r
55–99 U/L	95	67.49	66.51	0.072	−1.8	0.18
≥100 U/L	102	133.73	97.41	<0.001	−9.03	0.80

**Table 3:** Changes in GGT levels (ΔGGT = T2 − T1) across baseline GGT subgroups.

Baseline GGT Subgroups	*n*	Median ΔGGT
55–99 U/L	95	−2.58
≥100 U/L	102	−35.25

**Table 4:** Proportion of patients achieving GGT reduction.

Outcome	55–99 U/L (*n* = 95)	≥100 U/L (*n* = 102)
Reduction	67 (70.5%)	93 (91.2%)
No reduction	28 (29.5%)	9 (8.8%)

## Data Availability

The data presented in this study are available from the corresponding author upon reasonable request.

## Supplementary Materials

The following supporting information can be downloaded at: https://www.mdpi.com/article/10.3390/nu18111676/s1, Figure S1: Distribution of changes in serum GGT levels (ΔGGT = T2 − T1) according to baseline GGT subgroups (55–99 U/L and ≥100 U/L); Figure S2: Individual GGT trajectories between baseline and follow-up in participants with baseline GGT 55–99 U/L; Figure S3: Individual GGT trajectories between baseline and follow-up in participants with baseline GGT ≥ 100 U/L; Table S1: Commercial nutraceutical products used in the study; Table S2: Baseline distribution of alcohol use disorder severity according to DSM-5 classification across baseline GGT subgroups.

## Abbreviations

The following abbreviations are used in this manuscript:

ALT	Alanine aminotransferase
AST	Aspartate aminotransferase
AUD	Alcohol use disorder
CDT	Carbohydrate-deficient transferrin
DSM-5	Diagnostic and Statistical Manual of Mental Disorders, Fifth Edition
GGT	Gamma-glutamyl transferase
IQR	Interquartile range
PEth	Phosphatidylethanol

## References

[1] Corti A., Belcastro E., Dominici S., Maellaro E., Pompella A. (2020). The dark side of gamma-glutamyltransferase (GGT): Pathogenic effects of an “antioxidant” enzyme. Free Radic. Biol. Med..

[2] Vásquez-Tirado G.A., Nieto-Rivera S.M., Quispe-Castañeda C.V., Meregildo-Rodríguez E.D., Liñán-Díaz L.J., Guzmán-Aguilar W.M. (2025). Association between the levels of gamma-glutamyl transpeptidase and the risk of stroke: Systematic review and meta-analysis. Arq. Neuropsiquiatr..

[3] Neuman M.G., Malnick S., Chertin L. (2020). Gamma glutamyl transferase—An underestimated marker for cardiovascular disease and the metabolic syndrome. J. Pharm. Pharm. Sci..

[4] Ramandi A., George J., Behnoush A.H., Delavari A., Mohammadi Z., Poustchi H., Malekzadeh R. (2025). The association between serum gamma-glutamyl transferase and gastrointestinal cancer risk: A systematic review and meta-analysis. Cancer Med..

[5] European Association for the Study of the Liver (2018). EASL Clinical Practice Guidelines: Management of alcohol-related liver disease. J. Hepatol..

[6] Buechter M., Gerken G. (2022). Liver Function-how to screen and to diagnose: Insights from clinical studies and future perspectives. J. Pers. Med..

[7] Patel P.V., Flamm S.L. (2023). Alcohol-related liver disease including new developments. Clin. Liver Dis..

[8] Fakhari S., Waszkiewicz N. (2023). Old and new biomarkers of alcohol use: A narrative review. J. Clin. Med..

[9] Shetty A., Rahal K., Meza J., Saab S. (2026). Gastroenterologist’s guide to assess for alcohol use. Dig. Dis. Sci..

[10] de Bejczy A., Walther L., Nilsson-Wallmark C., Askerup B., Isaksson A. (2026). Decline of phosphatidylethanol (B-PEth) during abstinence in patients with alcohol use disorder undergoing withdrawal treatment, and the correlation of B-PEth with self-reported alcohol intake. Addiction.

[11] American Psychiatric Association (2013). Diagnostic and Statistical Manual of Mental Disorders.

[12] World Health Organization (2019). International Statistical Classification of Diseases and Related Health Problems (ICD-10).

[13] World Health Organization (2018). Global Status Report on Alcohol and Health.

[14] Bryazka D., Reitsma M., Griswold M., Abate K.H., Abbafati C., Abbasi-Kangevari M., Abbasi-Kangevari Z., Abdoli A., Abdollahi M., Abdullah A.Y.M. (2022). Population-level risks of alcohol consumption by amount, geography, age, sex, and year: A systematic analysis for the Global Burden of Disease Study 2020. Lancet.

[15] Ha Y., Jeong I., Kim T.H. (2022). Alcohol-related liver disease: An overview of pathophysiology, diagnosis and therapeutic perspectives. Biomedicines.

[16] Seitz H.K., Bataller R., Cortez-Pinto H., Gao B., Gual A., Lackner C., Mathurin P., Mueller S., Szabo G., Tsukamoto H. (2018). Alcoholic liver disease. Nat. Rev. Dis. Primers.

[17] Zhao L., Mehmood A., Yuan D., Usman M., Murtaza M.A., Yaqoob S., Wang C. (2021). Protective mechanism of edible food plants against alcoholic liver disease with special mention to polyphenolic compounds. Nutrients.

[18] Dukić M., Radonjić T., Jovanović I., Zdravković M., Todorović Z., Kraišnik N., Aranđelović B., Mandić O., Popadić V., Nikolić N. (2023). Alcohol, inflammation, and microbiota in alcoholic liver disease. Int. J. Mol. Sci..

[19] Newsome P.N., Cramb R., Davison S.M., Dillon J.F., Foulerton M., Godfrey E.M., Hall R., Harrower U., Hudson M., Langford A. (2018). Guidelines on the management of abnormal liver blood tests. Gut.

[20] Pinar-Sanchez J., Bermejo-Lopez P., Del Pozo J.S.G., Redondo-Ruiz J., Casado L.N., Andres-Pretel F., Bustillo M.L.C., Moreno M.E., Ruiz S.G., Santos J.J.S. (2022). Common laboratory parameters are useful for screening for alcohol use disorders: Designing a predictive model using machine learning. J. Clin. Med..

[21] Asrani S.K., Mellinger J., Sterling S., Lucey M.R., A Bradley K., Bhala N., Bray J., Chen P.-H., DiMartini A., Fernandez A. (2025). Reducing alcohol-associated liver disease burden in the general population. Lancet Gastroenterol. Hepatol..

[22] Nadkarni A., Massazza A., Guda R., Fernandes L.T., Garg A., Jolly M., Andersen L.S., Bhatia U., Bogdanov S., Roberts B. (2023). Common strategies in empirically supported psychological interventions for alcohol use disorders: A meta-review. Drug Alcohol Rev..

[23] Witkiewitz K., Litten R.Z., Leggio L. (2019). Advances in the science and treatment of alcohol use disorder. Sci. Adv..

[24] Kiluk B.D., Ray L.A., Walthers J., Bernstein M., Tonigan J.S., Magill M. (2019). Technology-delivered cognitive-behavioral interventions for alcohol use: A meta-analysis. Alcohol Clin. Exp. Res..

[25] Boness C.L., Witkiewitz K. (2023). Precision medicine in alcohol use disorder: Mapping etiologic and maintenance mechanisms to mechanisms of behavior change to improve patient outcomes. Exp. Clin. Psychopharmacol..

[26] Magill M., Apodaca T.R., Borsari B., Gaume J., Hoadley A., Gordon R.E.F., Tonigan J.S., Moyers T. (2018). A Meta-analysis of motivational interviewing process: Technical, relational, and conditional process models. J. Consult. Clin. Psychol..

[27] Mastrostefano A., Greco G., De Bacco C., Davini F., Polito G., Carnevale E., Anastasi G., Terracina S. (2025). Current pharmacotherapies for alcohol use disorder in Italy: From neurobiological targets to clinical practice. Targets.

[28] Jophlin L.L., Singal A.K., Bataller R., Wong R.J., Sauer B.G., Terrault N.A., Shah V.H. (2024). ACG Clinical Guideline: Alcohol-associated liver disease. Am. J. Gastroenterol..

[29] Field C.A., Richards D.K., Castro Y., Alonso Cabriales J., Wagler A., von Sternberg K. (2020). The effects of a brief motivational intervention for alcohol use through stages of change among nontreatment seeking injured patients. Alcohol Clin. Exp. Res..

[30] Bjørkhaug S.T., Aanes H., Neupane S.P., Bramness J.G., Malvik S., Henriksen C., Skar V., Medhus A.W., Valeur J. (2019). Characterization of gut microbiota composition and functions in patients with chronic alcohol overconsumption. Gut Microbes.

[31] Sun P., Li Y., Chang L., Tian X. (2019). Prognostic and clinicopathological significance of gamma-glutamyltransferase in patients with hepatocellular carcinoma: A PRISMA-compliant meta-analysis. Medicine.

[32] Lasserre A.M., Zhu Y., Kilian C., Llamosas-Falcón L., Rehm J., Probst C. (2025). Mediating role of psychological distress and alcohol use in socioeconomic disparities in deaths of despair: A causal mediation analysis using record linkage data. J. Epidemiol. Community Health.

[33] Müller C.P., Schumann G., Rehm J., Kornhuber J., Lenz B. (2023). Self-management with alcohol over lifespan: Psychological mechanisms, neurobiological underpinnings, and risk assessment. Mol. Psychiatry.

[34] Gan C., Yuan Y., Shen H., Gao J., Kong X., Che Z., Guo Y., Wang H., Dong E., Xiao J. (2025). Liver diseases: Epidemiology, causes, trends and predictions. Signal Transduct. Target. Ther..

[35] Ratner J.A., Blaney H., Rastegar D.A. (2024). Management of alcohol withdrawal syndrome in patients with alcohol-associated liver disease. Hepatol. Commun..

[36] Singal A.K., Vatsalya V., Agrawal R. (2024). Integrated multidisciplinary care model to manage the dual pathology of alcohol use disorder and of liver disease. Clin. Liver Dis..

[37] Eslam M., Sanyal A.J., George J., on behalf of the International Consensus Panel (2020). MAFLD: A consensus-driven proposed nomenclature for metabolic associated fatty liver disease. Gastroenterology.

[38] Hofmann S.G., Hayes S.C. (2019). The Future of Intervention Science: Process-based Therapy. Clin. Psychol. Sci..

[39] Ministry of Transport and Infrastructure, Romania (2025). Order No. 579/2025 on Mandatory Medical Examinations for Personnel with Safety-Related Duties in Transport.

[40] Gundermann K.J., Gundermann S., Drozdzik M., Mohan Prasad V.G. (2016). Essential phospholipids in fatty Liver: A scientific update. Clin. Exp. Gastroenterol..

[41] Gillessen A., Schmidt H.H. (2020). Silymarin as supportive treatment in liver diseases: A narrative review. Adv. Ther..

[42] Abenavoli L., Izzo A.A., Milic N., Cicala C., Santini A., Capasso R. (2018). Milk thistle (*Silybum marianum*): A concise overview on its chemistry, pharmacological, and nutraceutical uses in liver diseases. Phytother. Res..

