# Supplementary material for: Multimodal Nutraceutical and Psychological Intervention for GGT Reduction in Individuals with Alcohol Use Disorder
Source: Nutrients. 2026 May 23;18(11):1676. doi: 10.3390/nu18111676 (PMC13257675; doi:10.3390/nu18111676)
Supplement: Supplementary file 1 [file nutrients-18-01676-s001.zip › nutrients-4188233-supplementary.pdf]

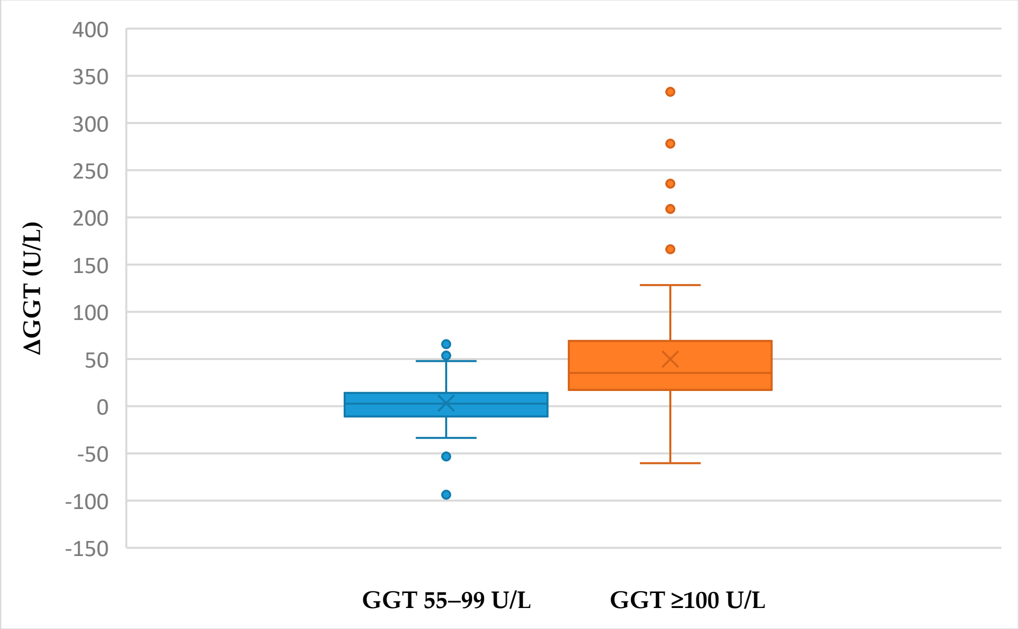

Figure S1: Distribution of changes in serum GGT levels (ΔGGT = T2 – T1) according to baseline GGT subgroups (55–99 U/L and ≥100 U/L)

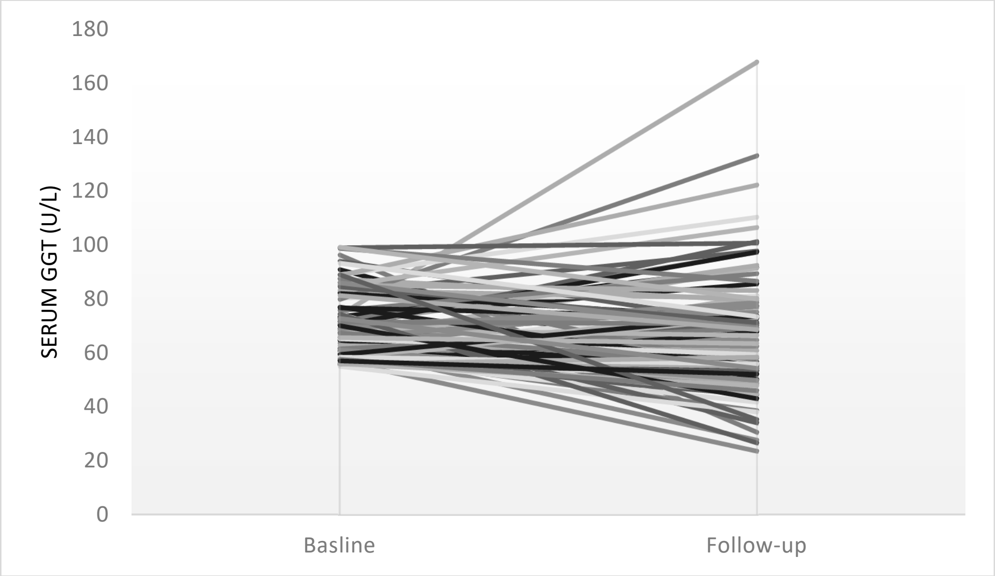

Figure S2: Individual GGT trajectories between baseline and follow-up in participants with baseline GGT 55–99 U/L

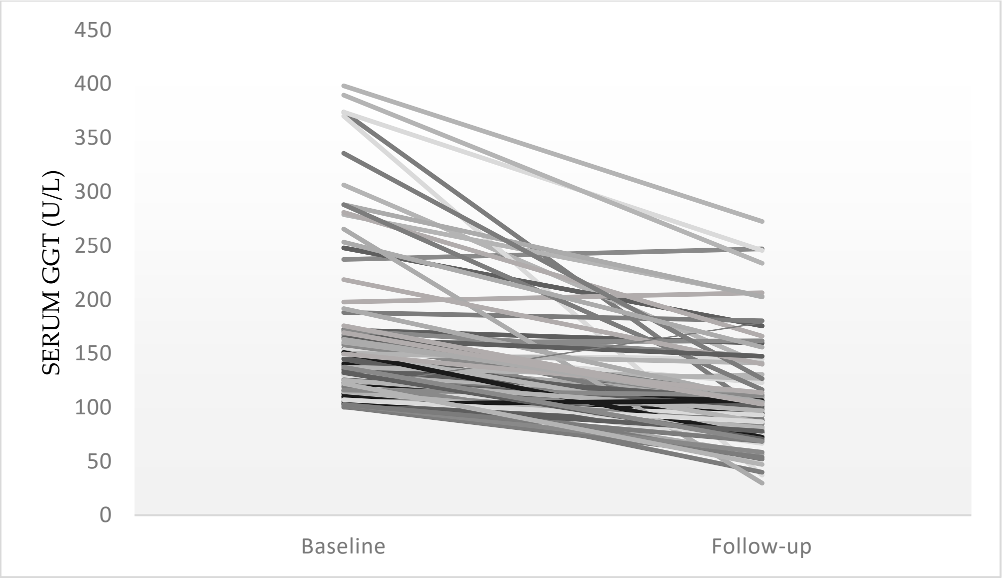

Figure S3: Individual GGT trajectories between baseline and follow-up in participants with baseline GGT ≥100 U/L

Table S1. Commercial nutraceutical products used in the study

Product	Active component
Lagosa®	Silymarin 150 mg
Liv.52®	Polyherbal antioxidant formulation
Essentiale Forte®	Essential phospholipids 300 mg

Table S2. Baseline distribution of alcohol use disorder severity according to DSM-5 classification across baseline GGT subgroups

AUD severity (DSM-5)	GGT 55–99 U/L (n = 95)	GGT ≥100 U/L (n = 102)	Total (N = 197)
Mild AUD	74 (77.9%)	2 (2.0%)	76 (38.6%)
Moderate AUD	16 (16.8%)	67 (65.7%)	83 (42.1%)
Severe AUD	5 (5.3%)	33 (32.4%)	38 (19.3%)
Total	95 (100%)	102 (100%)	197 (100%)

Note: Data are presented as n (%). AUD = alcohol use disorder; DSM-5 = Diagnostic and Statistical Manual of Mental Disorders, Fifth Edition; GGT = gamma-glutamyl transferase

Chi-square test of independence: $\chi^2(2) = 120.08$, $p < 0.001$.
